# Comparison of the Efficacy of Two Doses of Intranasal Atomized Dexmedetomidine as Premedication for Parental Separation Anxiety in Children Undergoing Elective Surgery

**DOI:** 10.7759/cureus.103239

**Published:** 2026-02-08

**Authors:** Devesh Kumar, Jeetendra K Bajaj, Sapna Bathla, Krishika Verma

**Affiliations:** 1 Department of Anesthesia and Intensive Care, Vardhman Mahavir Medical College (VMMC) and Safdarjung Hospital, New Delhi, IND

**Keywords:** children undergoing elective surgery, elective surgery, intranasal atomized dexmedetomidine, parental separation, premedication

## Abstract

Background

Preoperative anxiety in children is a common challenge associated with adverse perioperative outcomes, including poor parental separation, difficult mask acceptance, and emergence delirium. Dexmedetomidine, an alpha-2 adrenergic agonist, is increasingly explored as a noninvasive intranasal premedication.

Objective

This study compared the efficacy of two intranasal doses of dexmedetomidine (1 µg/kg vs. 2 µg/kg) in reducing separation anxiety and improving perioperative conditions in children undergoing elective surgery.

Methods

A prospective randomized controlled trial was conducted on 50 children aged between one and six years, American Society of Anesthesiologists (ASA) I-II, scheduled for elective surgery under general anesthesia. Participants were randomly assigned to receive either 1 µg/kg (Group I) or 2 µg/kg (Group II) of atomized intranasal dexmedetomidine 30 minutes before induction. Sedation Score (SS), Parental Separation Anxiety Score (PSAS), mask acceptance, hemodynamic parameters, postoperative pain (Face, Legs, Activity, Cry, Consolability (FLACC) scale), and emergence agitation (Emergence Agitation Scale (EAS)) were assessed.

Results

Group II demonstrated significantly better sedation at 30 minutes, a lower PSAS, improved mask acceptance, and reduced postoperative pain and emergence agitation (p<0.001). Hemodynamics remained stable in both groups without adverse events.

Conclusion

Intranasal dexmedetomidine 2 µg/kg provided superior preoperative sedation, smoother parental separation, better mask acceptance, and reduced postoperative discomfort compared with 1 µg/kg, without significant side effects.

## Introduction

The preoperative period is often highly distressing for children undergoing surgery. Anxiety during this phase is commonly triggered by fear of unfamiliar surroundings, medical personnel, parental separation, and invasive procedures such as injections [1]. Preoperative anxiety activates sympathetic, parasympathetic, and endocrine responses, leading to tachycardia, hypertension, and increased cardiac excitability, which may complicate anesthetic induction. Furthermore, heightened anxiety is associated with adverse postoperative outcomes, including emergence delirium, sleep disturbances, and long-term behavioral changes [2].

Several strategies have been employed to reduce preoperative anxiety in children, including preoperative counseling, parental presence during induction, and pharmacological premedication. Among these, sedative premedication remains the most reliable and effective method to reduce anxiety, minimize psychological trauma, and facilitate smooth induction of anesthesia [3].

An ideal pediatric premedication should have a rapid and predictable onset, short duration of action, easy and noninvasive administration, minimal side effects, adequate analgesic properties, and the ability to modulate autonomic responses. Dexmedetomidine, a highly selective alpha-2 adrenergic agonist, fulfills many of these criteria by providing sedation, anxiolysis, and analgesia without causing respiratory depression [4].

Premedication can be administered via oral, rectal, sublingual, intranasal, or inhalational routes. Although the oral route is widely used due to its simplicity and acceptance, its use is limited by delayed onset, unpleasant taste, gastrointestinal upset, prolonged recovery, and extensive first-pass hepatic metabolism resulting in low bioavailability (~16%). In pediatric patients, drug palatability further restricts the oral and sublingual routes.

Intranasal drug delivery has emerged as a safe, effective, and well-accepted alternative, offering rapid absorption and higher bioavailability (~65%) while avoiding venipuncture [5,6]. Optimal intranasal delivery requires small volumes (0.2-0.3 mL per nostril), as larger volumes are poorly absorbed. Atomized intranasal administration ensures uniform mucosal distribution and improved drug uptake.

Despite increasing use of intranasal dexmedetomidine in pediatric anesthesia, limited evidence exists comparing parental separation anxiety scores between different doses of atomized intranasal dexmedetomidine [7-24].

The primary objective of this study was to compare the effect of two doses of atomized intranasal dexmedetomidine (1 µg/kg and 2 µg/kg) on parental separation anxiety in children undergoing elective surgery. Secondary objectives included comparison of preoperative sedation, mask acceptance during induction, hemodynamic stability, postoperative pain, and emergence agitation between the two groups.

## Materials and methods

Study design and participants

A prospective, randomized, comparative study was conducted over a period of 18 months in the Department of Anesthesia at Safdarjung Hospital, New Delhi, India, after obtaining approval from the Institutional Ethics Committee of Vardhman Mahavir Medical College (VMMC) and Safdarjung Hospital (IEC/VMMC/SJH/Thesis/06/2022/CC-16 dated 11/07/2022) and registration with the Clinical Trials Registry of India (CTRI/2023/02/050003). The study commenced on 1st April 2023 and was completed on 15th September 2024. Written informed consent was obtained from the parents or legal guardians of all participating children.

Children were enrolled if they met the following inclusion criteria: age between one and six years, scheduled to undergo elective surgery under general anesthesia, classified as American Society of Anesthesiologists (ASA) physical status I or II, and having a normal airway assessment. Exclusion criteria included the presence of active upper respiratory tract infection, significant systemic organ dysfunction, known allergy or contraindication to dexmedetomidine, cardiac dysrhythmias or congenital heart disease, hemodynamic or respiratory instability, risk of airway obstruction, current use of psychotropic medications, or neurodevelopmental delay.

Sample size calculation

**Figure 1:**
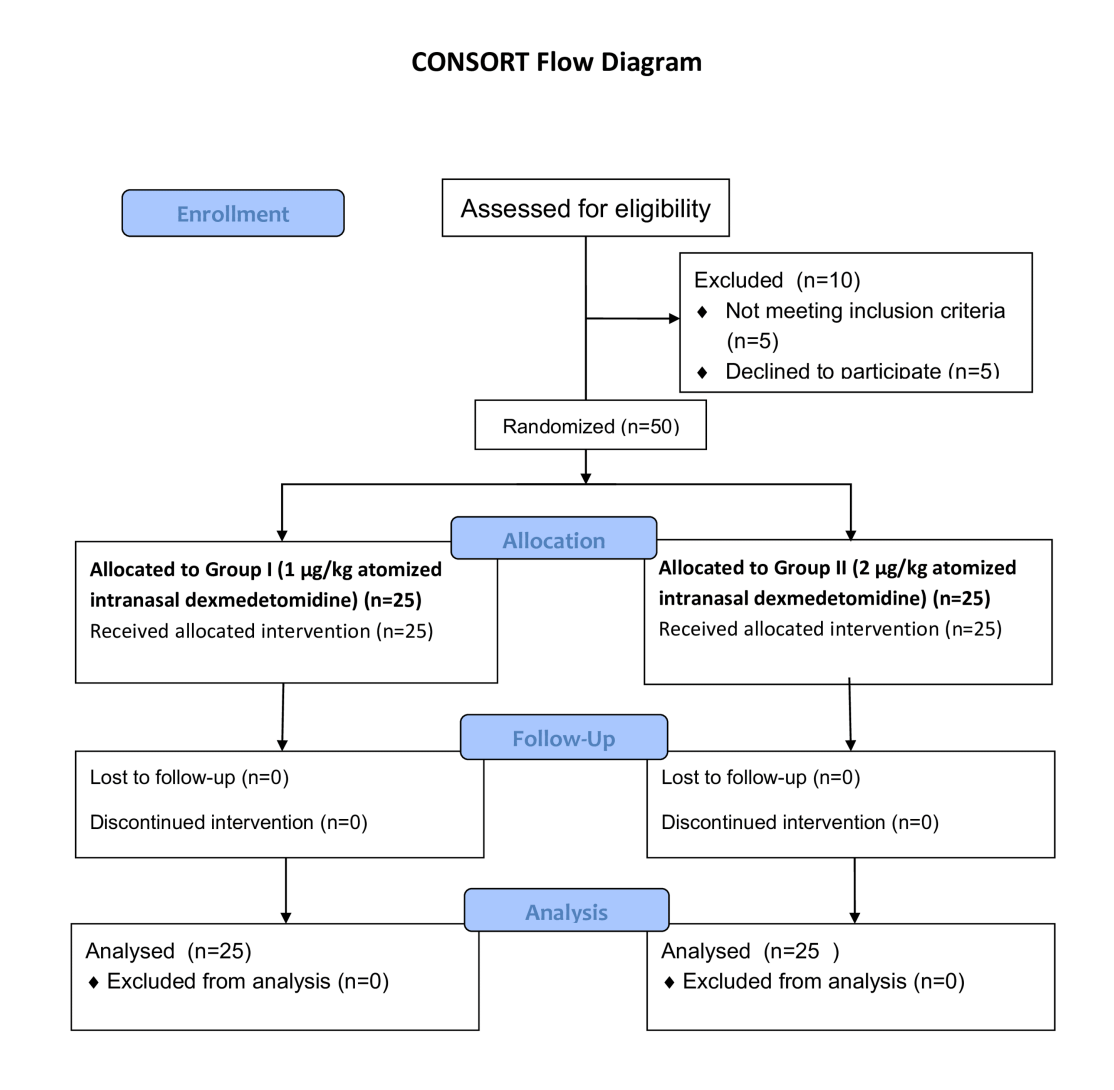
CONSORT flow diagram of participant enrollment, allocation, follow-up, and analysis µg/kg: micrograms per kilogram of body weight; n: number of participants; CONSORT: Consolidated Standards of Reporting Trials

A prospective randomized study by Yuen et al. [1] compared two intranasal doses of dexmedetomidine for pediatric premedication and reported satisfactory sedation (sedation score 3 or 4) in 29% of children receiving 1 µg/kg and 71% of those receiving 2 µg/kg. Based on these findings, with a study power of 90% and a significance level of 5%, the minimum required sample size was calculated to be 25 children in each group. As the study consisted of two groups, a total sample size of 50 children was included. All enrolled participants completed the study, and no dropouts or exclusions occurred after randomization. Participant flow is depicted in the Consolidated Standards of Reporting Trials (CONSORT) flow diagram (Figure 1). Randomization was performed using a computer-generated random number table for allocation. Allocation concealment was ensured using sequentially numbered, opaque, sealed envelopes prepared by an independent investigator not involved in patient assessment. Due to the nature of the intervention, the study was not double-blinded; however, outcome assessments were performed using standardized, validated scales.

Scales used

Ramsay Sedation Score (SS)

Sedation was assessed using the four-point sedation scale described by Ramsay et al. [25]. This scale grades the depth of sedation based on the child’s level of alertness and responsiveness. A score of 1 denotes an anxious, agitated, or restless child; a score of 2 indicates a cooperative, oriented, and tranquil child; a score of 3 represents a child who responds only to verbal commands; and a score of 4 denotes a child who is asleep with a brisk response to external stimuli. Higher scores indicate deeper levels of sedation, with scores ≥3 considered satisfactory sedation.

Mask Acceptance Scale

Mask acceptance during induction of anesthesia was evaluated using the four-point mask acceptance scale described by Wang et al. [26]. This scale assesses the child’s behavioral response to the application of the anesthesia face mask. A score of 1 represents poor acceptance, characterized by combative behavior, crying, or refusal of the mask. A score of 2 indicates fair acceptance, with moderate fear and mask acceptance only after persuasion. A score of 3 denotes good acceptance, with minimal fear and easy acceptance of the mask, while a score of 4 represents excellent acceptance, characterized by a calm, cooperative child who readily accepts the mask. Higher scores indicate better mask acceptance.

Parental Separation Anxiety Score (PSAS)

Parental separation anxiety was assessed using the four-point PSAS [27]. This scale evaluates the child’s emotional response at the time of separation from parents prior to induction of anesthesia. A score of 1 indicates a calm child who separates easily, a score of 2 reflects slight anxiety that is easily reassured, a score of 3 denotes moderate anxiety with crying but comforted, and a score of 4 represents severe anxiety characterized by persistent crying and clinging to parents. Higher scores indicate greater parental separation anxiety.

Emergence Agitation Scale (EAS)

Emergence agitation was assessed postoperatively using the four-point emergence agitation scale described by Watcha et al. [28]. A score of 1 indicates a calm child, a score of 2 represents a child who is not calm but can be easily consoled, a score of 3 denotes moderate agitation or restlessness, and a score of 4 reflects severe agitation with combative, disoriented, or thrashing behavior. Scores ≥3 were considered clinically significant emergence agitation, with higher scores indicating greater severity.

*Pain Assessment (Face, Legs, Activity, Cry, Consolability (FLACC)* *Scale)*

Postoperative pain was evaluated using the FLACC scale described by Merkel et al. [29]. This scale consists of five components: face, legs, activity, cry, and consolability, each scored from 0 to 2, resulting in a total score ranging from 0 to 10. A total score of 0 indicates no pain, scores of 1-3 indicate mild pain, scores of 4-6 indicate moderate pain, and scores of 7-10 indicate severe pain. Higher scores correspond to greater pain intensity.

Anesthetic technique

Preoperative fasting was ensured according to the ASA fasting guidelines: six hours for solid food and formula milk, four hours for breast milk, and two hours for clear liquids [30]. In the preoperative holding area, a baseline sedation score was recorded using a four-point sedation scale. Baseline vital parameters, including heart rate (HR), respiratory rate (RR), systolic blood pressure (SBP), diastolic blood pressure (DBP), and mean arterial pressure (MAP), oxygen saturation (SpO₂), and electrocardiography (ECG), were recorded and continuously monitored throughout the study.

Children received atomized intranasal dexmedetomidine at a dose of either 1 µg/kg or 2 µg/kg according to group allocation, administered 30 minutes prior to induction of anesthesia using a mucosal atomization device. Intranasal dexmedetomidine was administered using a mucosal atomization device (MAD), which delivers the drug as a fine mist to ensure uniform distribution over the nasal mucosa. A commercially available MAD, Teleflex® MAD Nasal™ (Teleflex Medical, Wayne, PA, USA), attached to a 1-mL syringe was used. The calculated dose was divided equally between both nostrils and administered to the child in a supine position with mild neck extension. Thirty minutes after drug administration, vital parameters were recorded in the preoperative room. Parental separation was then performed, and ease of separation was assessed using a four-point PSAS. Sedation scores were recorded at 5, 10, 20, and 30 minutes after administration of the study drug.

Intraoperative management

On arrival in the operating room, baseline intraoperative parameters were recorded. Anesthesia was induced using 8% sevoflurane in a 50% nitrous oxide-oxygen mixture. Intravenous access was established with a 22- or 24-gauge cannula, and fentanyl 2 µg/kg IV was administered. Propofol 1 mg/kg IV was given if required. After confirmation of adequate manual ventilation, neuromuscular blockade was achieved using vecuronium bromide 0.1 mg/kg IV.

A supraglottic airway device or appropriately sized endotracheal tube was inserted three minutes after administration of the muscle relaxant, following intermittent positive pressure ventilation with oxygen, nitrous oxide, and sevoflurane. Anesthesia was maintained with sevoflurane in oxygen and nitrous oxide (fraction of inspired oxygen (FiO₂) 0.3). Supplemental doses of fentanyl and vecuronium bromide were administered as required. Sevoflurane was discontinued five minutes prior to the anticipated end of surgery.

At the end of surgery, neuromuscular blockade was reversed using neostigmine 0.05 mg/kg IV and glycopyrrolate 0.01 mg/kg IV. All patients received 100% oxygen during emergence from anesthesia. The airway device was removed after pharyngeal suctioning when the child was fully awake and responding to verbal commands. Intraoperative parameters were documented throughout the procedure.

Postoperative assessment

Following surgery, children were transferred to the post-anesthesia care unit (PACU). Emergence agitation was assessed using the EA scale, postoperative pain was evaluated using the FLACC scale, and vital parameters, including HR, RR, non-invasive blood pressure (NIBP), and SpO₂, were recorded. All parameters were documented at 15-minute intervals for the first postoperative hour.

Statistical analysis

Data were entered into Microsoft Excel (Microsoft Corp., Redmond, WA, USA), and statistical analysis was performed using STATA version 15.0 (StataCorp LLC, College Station, TX, USA). Normality of continuous variables was assessed using the Shapiro-Wilk test. Categorical variables were expressed as frequencies and percentages, while continuous variables with normal distribution were presented as mean ± standard deviation. Comparisons between groups were performed using the unpaired t-test for continuous variables and the chi-square test for categorical variables. A p-value < 0.05 was considered statistically significant.

## Results

Baseline demographic variables, including age, weight, height (Table 1), ASA physical status, sex (Table 2), etc., were comparable between the two groups.

**Table 1:** Comparison of baseline demographic variables: age, height, and weight ^#^ The statistical significance of differences in mean values between Group I (1 µg/kg atomized intranasal dexmedetomidine) and Group II (2 µg/kg atomized intranasal dexmedetomidine) was analyzed using the unpaired t-test N: score; SD: standard deviation

Characteristics	Group I (N=25) Mean ± SD	Group II (N=25) Mean ± SD	t-value^#^	p-value
Age (years)	3.3 ± 1.6	3.4±1.5	−0.23	0.663
Height (centimeters)	97 ± 7.4	97.6±7.5	−0.28	0.832
Weight (kilograms)	13.2 ± 3.4	13.4±3.1	−0.22	0.879

**Table 2:** Comparison of baseline characteristics between groups: sex and ASA grading ^#^ The statistical significance of differences in proportions between Group I (1 µg/kg atomized intranasal dexmedetomidine) and Group II (2 µg/kg atomized intranasal dexmedetomidine) was analyzed using the chi-square test N: score; SD: standard deviation; ASA: American Society of Anesthesiologists

Characteristics	Group I (N=25) N (percentage)	Group II (N=25) N (percentage)	Chi-square value^#^	p-value
Gender: Male	17 (68%)	18 (72%)	0.094	0.775
Gender: Female	8 (32%)	7 (28%)		
ASA: Physical status I	14 (56%)	16 (64%)	0.334	0.662
ASA: Physical status II	11 (44%)	9 (36%)		

There was no statistically significant difference in preoperative SS between the groups at baseline and at 5, 10, and 20 minutes after administration of the study drug (Table 3). However, at 30 minutes, the sedation score was significantly higher in Group II compared to Group I (p < 0.001).

**Table 3:** Characteristics of participants as per the preoperative SS * A significant difference in mean values between Group I (1 µg/kg atomized intranasal dexmedetomidine) and Group II (2 µg/kg atomized intranasal dexmedetomidine) was analyzed using the unpaired t-test. SS: Sedation Score; SD: standard deviation; N: number of participants

Characteristics	Group I (N=25) Mean ± SD	Group II (N=25) Mean ± SD	t-value	p-value
Baseline SS	1±0	1±0	0.00	0.992
SS at 5 minutes	1±0	1.2±0.4	−2.50	0.829
SS at 10 minutes	1.4±0.5	1.9±0.5	−3.54	0.226
SS at 20 minutes	2.1±0.9	2.7±0.6	−2.78	0.163
SS at 30 minutes	2.2±1.0	3.1±0.8	−3.48	<0.001*

Statistically significant differences were observed between the two groups, with Group II demonstrating significantly lower PSAS and Mask Acceptance Scores compared to Group I (p < 0.001) (Table 4).

**Table 4:** Characteristics of participants as per the PSAS and the Mask Acceptance Score * A significant difference in mean values between Group I (1 µg/kg atomized intranasal dexmedetomidine) and Group II (2 µg/kg atomized intranasal dexmedetomidine) was analyzed using the unpaired t-test PSAS: Parental Separation Anxiety Score; SD: standard deviation; N: number of participants

Characteristics	Group I (N=25) Mean ± SD	Group II (N=25) Mean ± SD	t-value	p-value
PSAS	2.7±1.02	1.9±0.8	3.08	<0.001*
Mask Acceptance Score	2.9±1.2	2±0.9	3.01	<0.001*

Intraoperative HR, SBP, and DPB were significantly lower in Group II compared with Group I at all measured intervals (p < 0.05). Intraoperative MAP was also significantly lower in Group II at all time points except at 90 minutes, where no significant difference was observed. Intraoperative SpO₂ did not differ significantly between the two groups at any time point. There was also no statistically significant difference in the duration of anesthesia or recovery time between the groups (Table 5).

**Table 5:** Characteristics of participants as per the duration of anesthesia and recovery time * A statistical difference in mean values between Group I (1 µg/kg atomized intranasal dexmedetomidine) and Group II (2 µg/kg atomized intranasal dexmedetomidine) was analyzed using the unpaired t-test SD: standard deviation; N: number of participants

Characteristics	Group I (N=25) Mean ± SD	Group II (N=25) Mean ± SD	t-value	p-value
Duration of anesthesia in minutes	72.0±19.3	75.0±16.7	−0.60	0.252
Recovery time in minutes	9.6±1.6	9.6±1.3	0.00	0.992

Postoperatively, heart rate was significantly lower in Group II at all measured intervals, while no significant differences were observed in SBP, DBP, MAP, RR, or SpO_2_ (Table 6).

**Table 6:** Characteristics of participants as per the postoperative heart rate * A significant difference in mean values between Group I (1 µg/kg atomized intranasal dexmedetomidine) and Group II (2 µg/kg atomized intranasal dexmedetomidine) was analyzed using the unpaired t-test HR: heart rate; SD: standard deviation; N: number of participants

Characteristics	Group I (N=25) Mean ± SD	Group II (N=25) Mean ± SD	t-value	p-value
Baseline HR in beats/ minute	104.7±8.3	102.1±6.1	1.28	<0.001*
HR in beats/ minute at 15 minutes	104.7±7.5	101.5±4.7	1.78	<0.001*
HR in beats/ minute at 30 minutes	102.8±8.2	98.4±4.2	2.38	<0.001*
HR in beats/ minute at 45 minutes	100.5±8.4	95.8±3.5	2.62	<0.001*
HR in beats/ minute at 60 minutes	100.6±7.8	96.2±4.1	2.43	<0.001*

Postoperative pain assessed using the FLACC scale was significantly lower in Group II at baseline, 15 minutes, and 30 minutes, with no significant difference at 45 and 60 minutes (Table 7).

**Table 7:** Characteristics of participants as per the postoperative FLACC scale * A significant difference in mean values between Group I (1 µg/kg atomized intranasal dexmedetomidine) and Group II (2 µg/kg atomized intranasal dexmedetomidine) was analyzed using the unpaired t-test FLACC: Face, Legs, Activity, Cry, Consolability; SD: standard deviation; N: number of participants

Characteristics	Group I (N=25) Mean ± SD	Group II (N=25) Mean ± SD	t-value	p-value
Baseline FLACC	0.88±1.1	0.28±0.7	2.32	<0.001*
FLACC at 15 minutes	0.96±1.1	0.28±0.5	2.85	<0.001*
FLACC at 30 minutes	0.84±0.9	0.44±0.6	1.83	<0.001*
FLACC at 45 minutes	0.6±0.7	0.6±0.5	0.00	0.885
FLACC at 60 minutes	0.6±0.6	0.7±0.5	−0.64	0.773

The EAS was also significantly lower in Group II at baseline, 15 minutes, and 30 minutes, with no significant difference at later time points (Table 8).

**Table 8:** Characteristics of participants as per the postoperative EAS * A significant difference in mean values between Group I (1 µg/kg atomized intranasal dexmedetomidine) and Group II (2 µg/kg atomized intranasal dexmedetomidine) was analyzed using the unpaired t-test EAS: Emergence Agitation Scale; SD: standard deviation; N: number of participants

Characteristics	Group I (N=25) Mean ± SD	Group II (N=25) Mean ± SD	t-value	p-value
Baseline EAS	1.8±0.5	1.2±0.3	5.05	<0.001*
EAS at 15 minutes	1.6±0.4	1.2±0.4	3.54	<0.001*
EAS at 30 minutes	1.3±0.5	1.1±0.3	1.70	<0.001*
EAS at 45 minutes	1.2±0.4	1.3±0.5	−0.78	0.753
EAS at 60 minutes	1.2±0.4	1.3±0.5	−0.78	0.633

## Discussion

In the present study, no statistically significant difference was observed in preoperative SBP, DBP, MAP, or HR between the two groups after intranasal administration of the study drugs. Importantly, no episode of bradycardia or hypotension requiring medical intervention was noted in either group, indicating hemodynamic safety of both doses. Similar observations were reported in the study conducted by Agrawal et al. [11], supporting the cardiovascular stability associated with intranasal dexmedetomidine.

There was no statistically significant difference in preoperative sedation scores between the two groups at baseline and at earlier time intervals, except at 30 minutes after drug administration, when the sedation score was significantly higher in Group II compared to Group I. At the time of separation from parents, prior to transfer to the operating room, a statistically significant difference was observed in both parental separation anxiety score and sedation score, with children in Group II being calmer and better sedated. Similar findings were reported by Yuen et al. [1], Jin Xu et al. [10], and Agrawal et al. [11], all of whom demonstrated that dexmedetomidine administered at a dose of 2 µg/kg resulted in superior and more consistent sedation.

On entry into the operating room, children in Group II were more adequately sedated than those in Group I and demonstrated significantly better mask acceptance, which was evident both clinically and statistically. Comparable results were also observed in the study conducted by Agrawal et al. [11], reinforcing the beneficial role of higher-dose dexmedetomidine in facilitating smooth induction of anesthesia.

The difference in intraoperative HR, SBP, and DBP between the two groups at all measured intervals was statistically significant, with lower values observed in Group II. These findings are consistent with those reported by Schmidt et al. [17], who observed lower hemodynamic parameters in children premedicated with dexmedetomidine. Although dexmedetomidine is known to cause dose-dependent reductions in HR and BP, no episode of clinically significant bradycardia or hypotension requiring intervention was observed in our study, indicating that both doses were well tolerated.

In the present study, postoperative HR was significantly lower in Group II at all recorded intervals. Similar findings were reported by Elshafeey et al. [22], who studied 76 pediatric patients undergoing adenotonsillectomy and observed significantly lower postoperative heart rates in children receiving dexmedetomidine compared to those receiving ketamine at 10, 20, and 30 minutes postoperatively.

The difference in postoperative pain assessed using the FLACC scale was statistically significant between the two groups at most intervals, except at 45 and 60 minutes postoperatively. Dexmedetomidine administered at a dose of 2 µg/kg provided better postoperative analgesia compared to 1 µg/kg, which may contribute to a reduced requirement for postoperative opioid analgesics, thereby improving overall recovery quality.

In the present study, the difference in postoperative EAS scores between the two groups was statistically significant at all time intervals except at 45 and 60 minutes. These findings were consistent with those reported by Kim et al. [23], who evaluated 90 patients undergoing closed reduction of nasal bone fracture. In their study, patients receiving dexmedetomidine exhibited reduced incidence, duration, and severity of emergence agitation compared to the control group. Similar conclusions were also drawn by Shereef et al. [24], who compared nebulized dexmedetomidine, midazolam, and ketamine and found dexmedetomidine to be associated with the least emergence agitation.

Strengths of the study

The strengths of this study include its prospective randomized controlled design, which minimizes selection bias and enhances the internal validity of the findings. The use of validated and standardized assessment tools for sedation, parental separation anxiety, mask acceptance, postoperative pain, and emergence agitation ensured objective and reproducible outcome measurement. In addition, comprehensive perioperative monitoring allowed systematic evaluation of both efficacy and safety of intranasal dexmedetomidine at two different doses. The non-invasive intranasal atomized route further strengthens the clinical relevance of the study in pediatric anesthesia practice.

Limitations

This study has several limitations. It was conducted at a single tertiary care center, which may restrict generalizability. Only children with ASA physical status I-II undergoing elective surgeries were included, limiting applicability to sicker children, emergency procedures, or those with significant comorbidities. The study was not double-blinded, which may have introduced observer bias in the assessment of subjective outcomes such as sedation, parental separation anxiety, mask acceptance, and emergence agitation. Additionally, the type of elective surgical procedure was not standardized, which may have influenced postoperative analgesia outcomes.

## Conclusions

In children aged between one and six years with ASA physical status I-II undergoing elective surgical procedures under general anesthesia, intranasal dexmedetomidine at a dose of 2 µg/kg was more effective than 1 µg/kg in reducing parental separation anxiety, improving preoperative sedation and mask acceptance, and decreasing postoperative pain and emergence agitation. Both doses were well tolerated without clinically significant adverse effects. These findings support the use of intranasal dexmedetomidine as a safe and effective premedication in this specific pediatric population and clinical setting.
